# The Impact of Quarantines, Lockdowns, and ‘Reopenings’ on the Commercialization of Science: Micro and Macro Issues

**DOI:** 10.1111/joms.12692

**Published:** 2021-03-17

**Authors:** Donald S. Siegel, Maribel Guerrero

**Affiliations:** ^1^ Arizona State University; ^2^ Universidad del Desarrollo; ^3^ Northumbria University; ^4^ Lund University

**Keywords:** championing/leadership, commercialization of science, COVID‐19 pandemic, public‐private partnerships, scientific workplace, social networks, work‐life balance

## Abstract

In 2020, almost all research labs in industry, academia, and the government were shut down for long periods of time by political leaders to control the spread of the coronavirus. We consider the “micro” and “macro” implications of ongoing coronavirus disruptions in scientific research and the dissemination and commercialization of that research. We have identified three key unanswered research questions regarding these unprecedented disruptions: (1) How is the pandemic affecting conventional measures of scientific output (the quantity and quality of basic research) and performance, social networks, and the strategic management of innovation? (2) How is the pandemic affecting technology transfer offices, incubators, accelerators, science and technology parks, and other aspects of the innovation ecosystem? (3) How do pandemic disruptions affect micro‐level factors, such as role conflict, identity, work‐life balance, equity, diversity, inclusion, “championing,” leadership, and organizational justice?

## INTRODUCTION

2020 was the 40^th^ anniversary of two landmark pieces of legislation in the USA: the Bayh‐Dole and Stevenson‐Wydler Acts. These acts incentivized the commercialization of science at universities and federal/national labs, where most basic research is conducted. Such legislation and the concomitant rise of technology transfer at universities and federal/national labs inspired other nations to adopt similar laws (Guerrero and Urbano, 2019), resulting in a substantial increase in patenting, licensing, start‐ups, and collaborations with industry and entrepreneurs. Unfortunately, 2020 was also a year when almost all research labs in industry, academia, and government were shut down for long periods of time by political leaders to control the spread of the coronavirus.

Previous JMS’ COVID‐19 commentaries have focused mainly on the antecedents and consequences of firm responses to the pandemic. In contrast, we consider both ‘micro’ and ‘macro’ implications of ongoing coronavirus workplace disruptions, that is, quarantines, lockdowns, and re‐openings, on the scientific workplace at universities and federal/national labs. Such disruptions constitute fertile ground for theoretical and empirical research on the commercialization of science. We have identified some promising new avenues of research on this topic.

### Re‐Configuration of Production, Dissemination, and Commercialization of Science

According to Markman et al. (2008), the traditional scientific process combines knowledge modes and methods involving three dimensions: (1) The individual dimension related to the heterogeneity of entrepreneurial teams, experiences, and related incentives; (2) The organizational dimension related to corporate governance, relationships with trading partners or intermediaries, and boundary‐spanning activities; (3) The institutional dimension related to technological valuation and personal injury protection (PIP). The COVID‐19 pandemic has re‐configured this ‘traditional’ process as follows:

First, *scientific producers* have re‐oriented their priorities due to the pandemic, to focus on imminent threats to humanity. The pandemic has also led to re‐configuration of the ‘supply’ of basic research (digital scientific workplaces), new knowledge production modes (co‐production modes and new rules of the game related to intellectual property protection), demand for efficient scientific funding process (efficiency in review processes and allocation of funds), and an expansion of geographic scope (initially centred on the flow of information related to COVID‐19 from Chinese institutions and the emergence of global networks for responding to the pandemic).

An inability to conduct basic research may have enabled scientists to devote more attention to patenting, licensing, and start‐up formation relating to new or existing technologies, especially in the life sciences, where it is much more difficult to conduct non‐related COVID‐19 basic research remotely. For example, Kubota (2021) reports that some Stanford researchers who do not conduct COVID‐19 basic research are devoting more time to publications and technology transfer.

Second, *the scientific discovery/dissemination cycle* for scientists not engaged in coronavirus‐related basic and applied research has been interrupted or substantially curtailed during the COVID‐19 pandemic. Quarantines and lockdowns have shut down conferences, seminars, and other social events where knowledge is disseminated, collaboration occurs, funding sources are identified, and discoveries emerge. Also, many clinical trials and early stage research projects that could lead to life‐saving treatments worldwide have been abandoned due to lockdowns or because funding has been discontinued (AMRC, 2020). NPR reported that clinical trials for many important cancer drugs were interrupted (Lupkin, 2020). In the USA, non‐COVID‐19 research operations at universities, medical schools, and federal labs (most located in states with severe lockdowns) have also been shut down, leading to the cancellation of basic and applied research on cancer, heart disease, hypertension, Alzheimer’s disease, and other diseases that kill millions each year (WHO, 2020).

In contrast, we have also observed faster dissemination of scientific publications regarding discoveries related to coronavirus (treatments, vaccines, and studies of broader impacts) through virtual workshops and academic journals (where the editorial process usually takes more than 1–2 years). The thoughtful academic and policymakers’ debate focused on intellectual protection and commercialization mechanisms of current discoveries.

Third, *scientific commercialization related to the coronavirus* has flourished by transferring the discoveries to final users according to the national health organizations’ controls. In this phase, the main challenge has been full disclosure of all information related to coronavirus research and access to medicines that treat the virus. Pharmaceutical patents have restricted access to generic supplier companies. Consequently, the US federal government has attempted to secure exclusive rights to any vaccine created, while the German federal government has offered to pharmaceuticals to buy the rights to the vaccine. Other administrations have also revived the figure of compulsory licensing to facilitate access to vaccines, drugs, or technological devices. Nationalistic commercialization practices have promoted not only international cooperation but also international competition (WEF, 2021).

In contrast, we observe that although patent and trademark associations had modified their operational forms by introducing deadline extensions and fee waivers (see Amin‐Reimer and Christensen, 2020), *scientific commercialization unrelated to the coronavirus* appears to have languished (technology transfer offices are reporting that patent applications are down).

### Effects on Social Networks and Public‐Private Partnerships

Social networks involving ‘star’ scientists and their ‘offspring’ and collaborators have been shown to be important in the commercialization of science, in terms of stimulating patenting and start‐up creation. Networks of academic scientists who become entrepreneurs may also be important influences on the performance of such start‐ups. For instance, depending on their cooperation arrangements with other public and/or private bodies, the academic social network has been shown to have a favourably impact on the growth trajectory of start‐ups and their rate of survival. Differences in the embeddedness of academics in a network of ties external or internal to the university may be associated with different growth trajectories.

COVID‐19 has stimulated N‐Helix collaborations^1^ among the public sector, supply chain industrial actors, non‐profit organizations, and civil citizens. As a result, we have observed multiple collaborative initiatives in different stages (in preparation, pilots, demos, trials or ready) associated with the prevention (sanitation, automatization, vaccination), the diagnosis (telehealth diagnosis, bigdata, test kits), the treatment (medication, ventilators, medical devices), the information (databases, communications), and the life adaptation (education, work, shopping, metal health). Indeed, several grants or supporting initiatives have emerged for connecting entrepreneurs, innovators, investors, social media, and society. Contrarily, non‐related coronavirus researchers have temporarily paused existing or new collaboration agreements due to the government lockdown and quarantine uncertainty.

The best example of partnership disruption in the COVID‐19 era has been the landmark agreement between AstraZeneca and the University of Oxford for the coronavirus vaccine (known as ChAdOx1 nCoV‐19). This public‐private partnership enables worldwide development, manufacturing, and distribution of this vaccine, especially in low and middle‐income countries. It has been guaranteed by a longstanding relationship of trust and success to advance basic research between these scientific places (AstraZeneca, 2020). It explains the rapid configuration of this disruptive collaboration integrated by an extraordinarily talented team of scientists (representing the best tradition of research, teaching, and commercialization driving the university’s mission for centuries) and a new partner (Vaccitech).

### Effects on the Measurement of Research and Commercialization Performance

Scientific productivity is usually measured using three proxies: number of patents granted, number of products in development, and number of products on the market. Public‐private collaborations that enrolled ‘star’ scientists and ‘industrial’ scientists positively affect scientific productivity metrics. COVID‐19 will produce a disruption on the productivity metrics of scientific places that were ‘winners’ and ‘losers’. ‘Winners’ will show good indicators in terms of patents, innovations, markets as well as knowledge spill‐overs effects, and socio‐economic externalities. Contrarily, ‘losers’ will show a drop in productivity indicators. Future official statistics will undoubtedly offer insights about the resilience or decline effects of COVID‐19 on these scientific places across the globe.

### Effects of Coronavirus Restrictions on the Scientific Workplace‐Micro Issues

The pandemic is also affecting micro‐level factors that may be critical in the commercialization of research, such as role conflict, identity, work‐life balance, ‘championing’, and leadership (Balven et al., 2018). In this context, identity refers to the fact that when we encourage scientists to engage in the commercialization of their research, we are asking them to assume a new identity as an entrepreneur or, in some cases, to become public figures/celebrities (e.g., Neil Ferguson in the UK). This may lead to conflict with their traditional roles and identities as scientists and teachers.

Involvement in technology transfer also presents challenges, in terms of work‐life balance. In normal times, scientists may be interested in pursuing commercialization, but feel too time‐constrained to do so. Given that many children have (physically) out of school for 10 months, parents have had to devote more time to caring for their children at home. Another important individual‐level phenomenon is the role of leadership on the part of department chairs and senior faculty at universities and senior scientists at federal labs, which can involve inspiring junior scientists to engage in technology transfer. Leaders and managers can also ‘champion’ these activities through their own endeavours, and thus, serve as a role model for scientists who wish to engage in these activities. Alternatively, they may simply provide support (time allocation) for subordinates to pursue commercialization of their research. It is important for micro researchers to study how the pandemic is affecting the scientific workplace and the propensity of scientists to commercialize their research. We also need to learn more about how these ongoing scientific workplace disruptions are affecting research and commercialization.

### Promising New Avenues of Research

In sum, we have identified three new areas of micro and macro research on how the pandemic is affecting the commercialization of science:

1. How is the pandemic affecting conventional measures of scientific output (the quantity and quality of basic research) and performance, social networks, and the strategic management of innovation, including the commercialization of research? This includes an analysis of its effects on collaborative research and commercialization, including public‐private partnerships and other university‐industry collaborations.
2. How is the pandemic affecting innovation intermediaries and the entrepreneurial ecosystem, including technology transfer offices, incubators, accelerators, science and technology parks, and other aspects on the innovation ecosystem? This has important public policy implications, given that there is substantial public investment in property‐based institutions and high technology economic development initiatives.
3. We also need a better understanding of how the pandemic is affecting the scientific workforce, especially those who may potentially be involved in commercialization efforts. This means that management scholars should explore the importance of micro‐level factors, such as role conflict, identity, work‐life balance, ‘championing’, organizational justice, and leadership. Such research will help us determine how to better manage the scientific workforce and the process of research commercialization under trying conditions.

## Note

1 The triple helix model of innovation refers to a set of interactions between academia, government, and other organizational actors.
